# To Get Vaccinated, or Not to Get Vaccinated, That Is the Question: Illness Representations about COVID-19 and Perceptions about COVID-19 Vaccination as Predictors of COVID-19 Vaccination Willingness among Young Adults in The Netherlands

**DOI:** 10.3390/vaccines9090941

**Published:** 2021-08-24

**Authors:** Manja Vollmann, Christel Salewski

**Affiliations:** 1 Department of Socio-Medical Sciences, Erasmus School of Health Policy & Management, Erasmus University Rotterdam, 3000 DR Rotterdam, The Netherlands; 2 Department of Health Psychology, Faculty of Psychology, University of Hagen, 58097 Hagen, Germany; christel.salewski@fernuni-hagen.de

**Keywords:** Common Sense Model (CSM) of self-regulation, illness representations, Necessity-Concerns Framework (NCF), treatment perceptions, vaccination willingness

## Abstract

Mass vaccination is considered necessary to reduce the spread of COVID-19; however, vaccination willingness was found to be especially low among young adults. Therefore, based on the extended Common Sense Model, the unique effects and the interplay of illness representations about COVID-19 and perceptions about COVID-19 vaccination in explaining COVID-19 vaccination willingness was investigated using a cross-sectional design. An online survey measuring the relevant variables was filled in by 584 participants (69.9% female) between 18 and 34 years. Correlation analyses showed that all illness representation dimensions except from timeline and both dimensions of vaccination perceptions were related to vaccination willingness. The mediation analysis revealed that less personal control, more prevention control, more concerns about COVID-19 as well as more perceived necessity of and fewer concerns about the vaccination were directly related to higher vaccination willingness. Additionally, prevention control was indirectly related to higher vaccination willingness through stronger perceptions of necessity of the vaccination. The extended Common Sense Model proved to be useful in the context of illness prevention. Campaigns to improve vaccination rates should aim at increasing the perception that COVID-19 is preventable through vaccination and the personal need of the vaccination as well as at decreasing concerns about the vaccination.

## 1. Introduction

A growing body of evidence suggests that mass vaccination is a very efficacious measure to reduce the spread of COVID-19 infections in the current global pandemic [1,2,3]. A number of non-pharmacological measures introduced at the beginning of the pandemic, such as limitations in social contacts, closing public spaces, and increased personal hygiene, have been shown to be effective in reducing the infection rate and diminishing and postponing the peak number of infections [4], but were not sufficient in ending the pandemic. Accordingly, the infection rates remained high over the first year of the pandemic and large parts of Europe endured a third COVID-19 wave in early spring 2021 [5]. For example, according to the Dutch National Institute for Public Health and the Environment, there were approximately 50,000 new infections, more than 1500 new hospital admissions, of which about 300 were in intensive care units, and about 175 deaths due to COVID-19 per week at that time in the Netherlands [6]. These high rates and the consequential prolongation of the lockdown measures had and still have tremendous negative effects on health care processes [7], people’s mental and physical health [8,9], and the economy [10].

Since December 2020, several vaccines against COVID-19 are available [11]. Mass COVID-19 vaccination campaigns have been launched in many countries since then to protect individuals and, even more important, to achieve herd immunity. Herd immunity is defined as “… the indirect protection from infection conferred to susceptible individuals when a sufficiently large proportion of immune individuals exist in a population” [12] (p. 737), resulting in a reduction or even elimination of disease transmission. The percentage of people that needs to be immune against COVID-19 in order to achieve herd immunity is estimated at about 60% to 75% [12,13,14,15,16]. As reaching this threshold through natural infections is not desirable [14,15,16], about 75% to 90% of the population is required to be vaccinated against COVID-19, depending on the efficacy of the COVID-19 vaccines [13].

Consequently, a crucial prerequisite of attaining herd immunity is the willingness of the individuals in a population to receive the vaccine. At the beginning of the pandemic, Neumann-Böhme and colleagues [14] concluded that the level of willingness to receive the COVID-19 vaccine might not be sufficient to reach herd immunity in various European countries, including the Netherlands. The results of two large Dutch studies showed that the percentage of people living in the Netherlands willing to receive the COVID-19 vaccine increased during the course of the pandemic (from about 60–65% in summer/autumn 2020 up to about 86% in spring 2021) [17,18]. However, international studies indicate that the willingness to receive the COVID-19 vaccine considerably depends, among other socio-demographic characteristics such as gender and education, on age, with the lowest levels of vaccination willingness found among young adults [14,19,20]. In the Netherlands, the percentage of adults between 18 and 34 years who are willing to receive the COVID-19 vaccine constantly lies about 10 percentage points below the average percentage of the whole population [17,18]. These findings indicate that young adults are less likely to add to the percentage of vaccinated individuals required for herd immunity, while contributing the most to the transmission of COVID-19 [21], probably due to an overall lower adherence to social distancing measures [22,23]. Therefore, identifying factors and processes that determine the willingness to receive the COVID-19 vaccine among young adults is highly valuable in order to develop effective interventions that promote vaccination willingness to eventually defeat COVID-19 [24,25].

### 1.1. Illness Representations about COVID-19 and Perceptions about COVID-19 Vaccination as Determinants of the Willingness to Receive the COVID-19 Vaccine

The extended Common Sense Model, combining the Common Sense Model of self-regulation (CSM) [26] and the Necessity-Concerns Framework (NCF) [27], qualifies as a suitable theoretical approach to investigate vaccination willingness in the targeted group of young adults as it describes general psychological factors and processes that are involved in the (pharmacological) management of current and future health threats.

Within the Common Sense Model of self-regulation, illness representations are specified as important determinants of illness-related behaviors that aim at enhancing health, preventing and controlling illness, and rehabilitating from illness. Illness representations are subjective beliefs and emotions about an illness that are formed by any individual after recognizing a (potential) health threat. Cognitive representations contain beliefs about the (number of) symptoms attributed to the illness (“identity”), about the duration (“timeline”), consequences, and causes of the illness, and about the possibilities to prevent, control, and cure the illness (“control”). Emotional representations refer to the mostly negative emotions elicited by the illness, such as concern, fear, and upset [26,28,29]. Finally, an overriding dimension refers to whether a person has a coherent picture of the illness (“understanding”) [30].

Previous empirical studies showed that various illness representation dimensions are significantly associated with illness preventive behaviors, including vaccination. Particularly, perceptions of more severe consequences and possibilities for prevention, more concerns and worries about the illness, and a better understanding of the illness were positively related to the intention to and the actual engagement in preventive behaviors [31,32,33,34,35,36,37,38,39,40,41,42,43]. Neto and colleagues [44] investigated the associations of illness representations about COVID-19 with non-pharmacological preventive behaviors and found that more concerns about COVID-19 and more perceived personal control about COVID-19 were related to more social distancing, handwashing, and self-isolation.

The Necessity-Concerns Framework specifically zooms in on treatment perceptions as determinants of illness behaviors involving the use of pharmaceuticals. Treatment perceptions regarding pharmaceuticals are divided into necessity perceptions which refer to the perceived personal need for the pharmaceuticals in order to prevent the (deterioration of the) illness and concerns about side effects and negative long-term consequences of the pharmaceuticals [27,45].

Former empirical studies found evidence that both dimensions of treatment perceptions are significantly related to the uptake of and adherence to pharmaceuticals, including vaccination. In particular, stronger perceptions of necessity and fewer concerns are associated with higher intentions to and actual higher levels of medication/vaccination uptake and adherence [34,37,39,46,47,48,49,50,51].

Horne [27] combined the CSM and the NCF by suggesting that perceptions of necessity of a specific pharmaceutical are mainly determined by illness representations, in particular representations about the seriousness of the illness. Empirical studies indeed showed that perceptions of severe symptoms, a long duration, and many adverse consequences stimulate perceptions of necessity. Additionally, it was found that stronger perceptions of the effectiveness of the pharmaceutical in controlling the illness or its onset, stronger emotional representations, and a better understanding of the illness are related to stronger necessity perceptions [47,52,53,54,55,56,57]. On the other hand, concerns about a specific pharmaceutical are not likely to be influenced by illness representations, but are rather informed by general beliefs about the nature of medicines or by past experiences with particular medicines than by illness representations [27].

The assumption of Horne [27] that illness representations determine perceptions of necessity (but not concerns) suggests mediating processes. In particular, it implies that illness representations are related to the use of a certain pharmaceutical through perceptions of necessity of this pharmaceutical. To our knowledge, no previous research has fully and properly investigated these mediating processes. The findings of Wilhelm and colleagues [57] indicate that weaker beliefs about personal control and stronger beliefs about treatment control are related to better medication adherence through stronger necessity perceptions. However, these mediation effects were not statistically tested and the other illness representation dimensions were not included. Another study focused more globally on health locus of control and found an indirect effect of a strong locus in powerful others on adherence through stronger necessity perceptions [58].

### 1.2. The Present Study

The overall aim of the present study was to investigate factors and processes explaining the willingness to receive the COVID-19 vaccine among young adults in the Netherlands based on the extended Common Sense Model. Vaccination willingness instead of vaccination uptake was chosen as outcome measure because young adults were not yet entitled to receive a COVID-19 vaccine during the time of the study.

Firstly, the unique impact of illness representations about COVID-19 and perceptions about COVID-19 vaccination (vaccination perceptions) on the willingness to receive the COVID-19 vaccine was examined. Based on the CSM [26] and the NCF [27] as well as previous study results, it was expected that the dimensions of both concepts are significantly related to vaccination willingness.

Secondly, the associations between illness representations of COVID-19 and perceptions of necessity of COVID-19 vaccination were explored. Based on the assumptions of Horne [27] and previous empirical findings, it was hypothesized that illness representations of COVID-19 are significantly associated with perceptions of necessity of COVID-19 vaccination.

**Figure 1:**
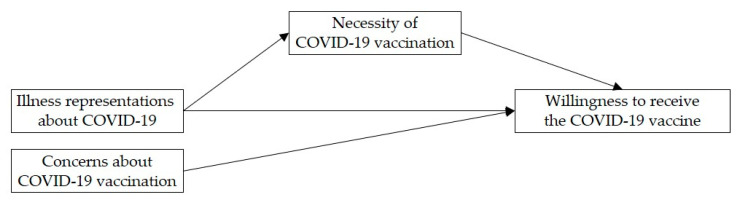
Theoretical model in which perceptions of necessity of COVID-19 vaccination (but not concerns about COVID-19 vaccination) mediate the relationship between illness representations about COVID-19 and the willingness to receive the COVID-19 vaccine.

Finally, the interplay of illness representations about COVID-19 and perceptions about COVID-19 vaccination in explaining the willingness to receive the COVID-19 vaccine was studied. Based on the assumptions of Horne [27], it was tested whether perceptions of necessity of COVID-19 vaccination (but not concerns) mediate the relationship between illness representations about COVID-19 and COVID-19 vaccination willingness (see Figure 1).

## 2. Materials and Methods

### 2.1. Procedure and Participants

Participants were recruited during the third COVID-19 wave in the Netherlands between 22 March and 10 May 2021 via posts on social media (e.g., Facebook, Instagram, LinkedIn, WhatsApp), including the request to pass the information on. Young adults between 18 and 34 years who have not been vaccinated against COVID-19 were invited to take part in this online study that was programmed in Questback’s survey software Unipark. After opening the link to the questionnaire, participants had to complete an informed consent that emphasized voluntary participation and anonymity. The study followed the principles of the Declaration of Helsinki and participants were treated according to the American Psychological Association ethical standards. In order to avoid missing data, all items were mandatory and could not be skipped. Participants received no compensation for participation. On average, it took 6 min to fill in the questionnaire.

Of the 752 persons who started the questionnaire, those who gave no informed consent (*n* = 12), were older than 34 years (*n* = 7), or had already been vaccinated (*n* = 65) were screened out immediately. Participants who did not fully complete the questionnaire (*n* = 68) or completed it in an unreasonable time frame (*n* = 16) were excluded. This resulted in a sample of 584 participants with a mean age of 25 years (*SD* = 3.64, range 18–34 years). The sample was highly educated. Most of the participants were employed or were in education. The majority of the participants indicated not to be at higher risk of severe COVID-19 due to an underlying health condition. A large share of the sample reported not having been infected with COVID-19 so far, but knowing someone who had already been infected with COVID-19. See Table 1 for a detailed sample description.

### 2.2. Measures

The questionnaire was administered in Dutch. After assessing socio-demographic information, COVID-19 vaccination willingness, illness representations about COVID-19, and perceptions about COVID-19 vaccination were measured. Means and standard deviations for all measures can be found in Table 2.

*Willingness to receive the COVID-19 vaccine*. The willingness to receive the COVID-19 vaccine was measured with the single item “Do you plan to get vaccinated against COVID-19 as soon as you receive an invitation?”. Responses were given on a 11-point scale ranging from 0 = definitely not to 10 = definitely yes, with higher scores indicating higher COVID-19 vaccination willingness.

*Illness representations about COVID-19*. A modified version of the Brief Illness Perception Questionnaire (Brief IPQ) [53,59] was used to measure the illness representations about COVID-19. Following the recommendation of Broadbent et al. [53] to adapt the Brief IPQ to specific illnesses, the word “illness” was replaced with ”COVID-19” in all items. Additionally, as in the IPQ-R for healthy people (IPQ-RH) [33], the wording of the items was adapted so that they can also be answered by people who have not been infected by COVID-19. Moreover, the items focused on COVID-19 among young adults.

All illness representation dimensions were measured with single-items that could be answered on a 11-point Likert scale ranging from 0 to 10. The Brief IPQ measures the five cognitive dimensions of identity, timeline, consequences, personal control, and treatment control. For example, the consequences dimension was assessed with the item “How much does COVID-19 affects the life of an infected young adult?” with 0 = not at all to 10 = very much. Following Vollmann et al. [42], prevention control was added as an additional cognitive dimension. Two items were used to measure prevention through own behavior, i.e., “How much do you think a young adult can prevent getting COVID-19 by his/her own behavior?”, and prevention through vaccination, i.e., “How much do you think vaccination is effective in the fight against COVID-19?”. The Brief IPQ further measures the two emotional illness representation dimensions, concern and emotional response, as well as illness understanding. Higher scores indicate stronger perceptions that COVID-19 involves many complaints, has a long duration, severely affects the life of an infected young adult, can be controlled by own behavior or medical treatment, can be prevented by own behavior or vaccination, elicits concerns, produces negative emotions, and is understandable.

*Perceptions about COVID-19 vaccination*. The necessity of and concerns about COVID-19 vaccination were operationalized by two subscales of the Vaccination Attitudes Examination scale (VAX scale) [60], i.e., mistrust of vaccine benefit (=necessity, reversely coded) and worries about unforeseen future effects (=concerns). Both subscales contain three items that were reworded to refer specifically to vaccination against COVID-19, e.g., “I worry about the unknown effects of COVID-19 vaccination in the future”. Responses were given on a 6-point scale ranging from 0 = strongly disagree to 5 = strongly agree. Items were averaged with higher scores indicating higher perceptions of necessity (α = 0.91) and more concerns (α = 0.83).

### 2.3. Data Analysis

**Figure 2:**
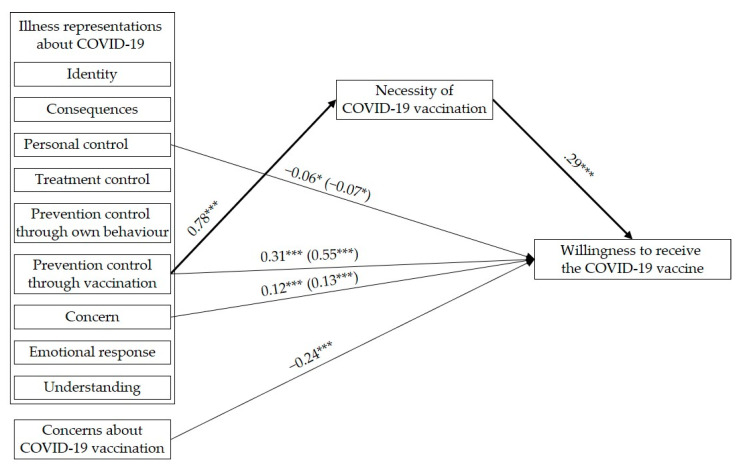
Results of the multiple mediation analysis. Only significant paths are displayed for figure clarity. Coefficients in parentheses represent total effects. Standardized coefficients are reported. Significant indirect effects are indicated by bold printed paths. * *p* < 0.05, *** *p* < 0.001.

Statistical analyses were performed with SPSS 27 and Mplus 5.2. First, bivariate associations between the study variables were examined by Pearson correlation analyses. As none of the background variables—i.e., age, gender, belonging to the risk group, having been infected with COVID-19, and having someone in the social network who has been infected with COVID-19—were significantly related to vaccination willingness (*r*s ≤ |0.07|, *p*s ≥ 0.10), they were not included as control variables in the subsequent analyses. Secondly, in order to examine the unique effects of illness representations about COVID-19 and perceptions about COVID-19 vaccination on COVID-19 vaccination willingness, a multiple regression analysis was performed. To keep the number of predictors to a minimum, only illness representation dimensions and vaccination perception dimensions that were significantly correlated with vaccination willingness were included. Thirdly, in order to examine the unique effects of illness representations about COVID-19 on perceptions of necessity of COVID-19 vaccination, a multiple regression analysis was performed. To keep the number of predictors to a minimum, only illness representation dimensions that were significantly correlated with perceptions of necessity of COVID-19 vaccination were included. Finally, the model presented in Figure 1 was tested by a mediation analysis using path analysis based on maximum likelihood estimation. Again, in order to estimate the most parsimonious model, only illness representation dimensions that were significantly correlated with perceptions of necessity or vaccination willingness were included (see Table 4 and Figure 2). The indirect effects of the independent variables (illness representation dimensions) on the dependent variable (vaccination willingness) via the mediator (perceptions of necessity) were estimated by bootstrapping with 10,000 bootstrap samples as recommended by Hayes [61]. All coefficients are reported in standardized form.

## 3. Results

### 3.1. Associations of Illness Representations about COVID-19 and Perceptions about COVID-19 Vaccination with the Willingness to Receive the COVID-19 Vaccine

The results of the correlation analyses (see Table 2) indicate that, except for timeline, all illness representation dimensions and both vaccination perception dimensions were significantly associated with the willingness to receive the vaccine. In particular, more symptoms attributed to a COVID-19 infection, more serious perceived consequences of a COVID-19 infection, weaker beliefs that a COVID-19 infection can be controlled by own behavior, stronger beliefs that a COVID-19 infection can be medically treated, more confidence that a COVID-19 infection can be prevented through own behavior and through vaccination, more concerns about COVID-19, stronger emotional responses to COVID-19, and a better understanding of COVID-19 were related to higher vaccination willingness. Moreover, stronger perceptions of necessity of and fewer concerns about COVID-19 vaccination were also related to a higher willingness to receive the COVID-19 vaccine.

The multiple regression analysis (see Table 3, middle column) revealed that all illness representation dimensions and vaccination perception dimensions together explained 69% of the variance in vaccination willingness. Three illness representation dimensions and both vaccination perception dimensions were significantly uniquely related to vaccination willingness. In particular, weaker beliefs that a COVID-19 infection can be controlled by own behavior, stronger perceptions that a COVID-19 infection can be prevented through vaccination, and more concerns about COVID-19 as well as stronger perceptions of necessity of and fewer concerns about COVID-19 vaccination were related to a higher willingness to receive the COVID-19 vaccine. With respect to the effect size, the effect of personal control is negligible, the effect of concerns about COVID-19 is rather small, and the effects of prevention control through vaccination, perceptions of vaccination necessity, and concerns about the vaccination are small to medium.

### 3.2. Associations of Illness Representations about COVID-19 with Perceptions of Necessity of COVID-19 Vaccination

The results of the correlation analyses (see Table 2) indicate that, except for timeline, all illness representation dimensions were significantly correlated with the perceptions of necessity of COVID-19 vaccination. In particular, more symptoms attributed to a COVID-19 infection, more serious perceived consequences of a COVID-19 infection, weaker beliefs that a COVID-19 infection can be controlled by own behavior, stronger beliefs that a COVID-19 infection can be medically treated, more confidence that a COVID-19 infection can be prevented through own behavior and through vaccination, more concerns about COVID-19, stronger emotional responses to COVID-19, and a better understanding of COVID-19 were related to stronger perceptions of necessity of COVID-19 vaccination.

The multiple regression analysis (see Table 3, right column) revealed that all illness representation dimensions together explained 66% of the variance in the perceptions of necessity of COVID-19 vaccination. Only one illness representation dimension was significantly uniquely related to the necessity perceptions, i.e., stronger perceptions that a COVID-19 infection can be prevented through vaccination was related to stronger perceptions of necessity of COVID-19 vaccination. This effect can be considered large in size.

### 3.3. Direct and Indirect Effects of Illness Representations about COVID-19 via Perceptions of Necessity of COVID-19 Vaccination and Direct Effects of Concerns about COVID-19 Vaccination on the Willingness to Receive the COVID-19 Vaccine

The results of the mediation analysis are presented in Table 4 and Figure 2 (only depicting the significant paths of the model). The analysis revealed significant direct effects of the illness representation dimensions personal control, prevention control through vaccination, and concerns about COVID-19 as well as both vaccination perception dimensions on vaccination willingness. These direct effects indicate that weaker beliefs that a COVID-19 infection can be controlled by own behavior, stronger perceptions that a COVID-19 infection can be prevented through vaccination, and more concerns about COVID-19 as well as stronger perceptions of necessity of and fewer concerns about COVID-19 vaccination are related to a higher willingness to receive the COVID-19 vaccine. Again, with respect to the effect size, the effect of personal control is negligible, the effect of concerns about COVID-19 is rather small, and the effects of prevention control through vaccination, perceptions of vaccination necessity, and concerns about the vaccination are small to medium. Additionally, a significant positive indirect effect of the dimension prevention control through vaccination on vaccination willingness via necessity perceptions, 0.23, BC 95% CI [0.15, 0.32], was found. This indirect effect indicates that stronger perceptions that a COVID-19 infection can be prevented through vaccination are related to stronger perceptions of necessity of COVID-19 vaccination, which, in turn, is associated with a higher willingness to receive the COVID-19 vaccine.

**Table 4:** Results of the multiple mediation analysis.

Predictors	Outcome			
	Perceptions of Necessity of the Vaccination	Willingness to Receive the Vaccine (Direct Effect)	Willingness to Receive the Vaccine (Total Effect)	Indirect Effect ^a^ via Necessity
	ß	ß	ß	ß (BC 95% CI)
**Illness representations**				
Identity	−0.01	0.02	0.01	−0.00 (−0.02, 0.01)
Consequences	−0.02	0.05	0.04	−0.01 (−0.02, 0.01)
Personal control	−0.03	−0.06 *	−0.07 *	−0.01 (−0.02, 0.01)
Treatment control	0.01	0.04	0.05	0.00 (−0.01, 0.02)
Prevention control through own behavior	0.03	0.04	0.04	0.01 (−0.01, 0.03)
Prevention control through vaccination	0.79 ***	0.31 ***	0.55 ***	0.23 (0.15, 0.32)
Concern	0.02	0.12 ***	0.13 ***	0.01 (−0.01, 0.03)
Emotional response	0.02	−0.00	0.00	0.01 (−0.01, 0.02)
Understanding	0.03	−0.03	−0.02	0.01 (−0.01, 0.02)
**Treatment perceptions**				
Necessity	-	0.29 ***	-	-
Concerns	-	−0.24 ***	-	-

Note. The multiple mediation analysis was calculated with illness representation dimensions about COVID-19 and concerns about COVID-19 vaccination as independent variables, perceptions of necessity of COVID-19 vaccination as mediator of the illness representation dimensions, and COVID-19 vaccination willingness as dependent variable. ^a^ Indirect effects are significant at *p* < 0.05 when zero is not included in the bias corrected 95% confidence interval (BC 95% CI). * *p* < 0.05, *** *p* < 0.001.

## 4. Discussion

The present study investigated factors and processes determining the willingness to receive the COVID-19 vaccine. More specifically, based on the extended Common Sense Model, the interplay between illness representations about COVID-19 and perceptions about COVID-19 vaccination in explaining vaccination willingness among young adults in the Netherlands was examined. The results may be informative for developing public health campaigns to change relevant psychological determinants [24], which may result in higher vaccination rates.

As expected, all illness representation dimensions except for timeline and both vaccination perception dimensions were significantly associated with COVID-19 vaccination willingness. These findings are in line with the CSM [26,28,29] and previous research that identified representations about an illness as important determinants of illness-preventive behaviors, including vaccination willingness and uptake [34,37,39,41,43]. The findings are also in accordance with the NCF [27] and empirical findings indicating that perceptions about a specific pharmaceutical determine the uptake of and adherence to this pharmaceutical, including vaccination willingness and uptake [37,39,49,50,51].

Among the illness representations, the dimensions prevention control through vaccination and concern appeared to be of special importance as they showed meaningful unique effects on the willingness to receive the COVID-19 vaccine. After controlling for all other illness representation dimensions and the two vaccination perception dimensions, stronger perceptions that a COVID-19 infection can be prevented through vaccination and more concerns about COVID-19 were related to a higher vaccination willingness. This confirms previous research showing quite consistently that confidence in the effectiveness of preventive measures and negative emotions or concerns about an illness are important predictors of illness-preventive behaviors [31,32,34,36,37,41,42,43]. Additionally, both vaccination perception dimensions, i.e., perceptions of necessity of and concerns about COVID-19 vaccination, showed meaningful unique effects on COVID-19 vaccination willingness. After controlling for all illness representation dimensions and the respective other vaccination perception dimension, a stronger perceived personal need for the vaccination in order to prevent a COVID-19 infection was related to higher vaccination willingness, while more concerns about side effects and long-term consequences of the vaccination were associated with lower vaccination willingness. These results replicate earlier findings indicating that the two treatment perception dimensions are independently related to the uptake of and adherence to pharmaceuticals [47,57,62,63]. These findings also correspond with systematic reviews on determinants of COVID-19 vaccination hesitancy [19,64,65] and the uptake of seasonal and pandemic influenza vaccination [66,67] that highlighted, in addition to a number of contextual factors, lower risk perceptions and worry about the disease as well as concerns about the effectiveness and safety of the vaccine as important barriers of vaccination intention and uptake.

In line with the expectations based on Horne’s [27] assumption and previous results [47,52,53,54,55,56,57], all illness representation dimensions except for timeline were significantly related to perceptions of the necessity of COVID-19 vaccination. The pattern of the bivariate associations suggests that negative illness representations in terms of illness seriousness and emotional impact as well as positive illness representations in terms of treatment control and prevention control go together with the perception of a personal need for COVID-19 vaccination. Interestingly, only the dimension prevention control through vaccination was uniquely related to necessity perceptions, indicating that the perception that a COVID-19 infection can effectively be prevented by vaccination might trigger the perception that protecting and maintaining one’s health depends on the vaccination.

Most importantly, as hypothesized, the present findings suggest that the effect of prevention control on vaccination willingness is partially mediated by necessity perceptions. Accordingly, stronger perceptions that a COVID-19 infection can be prevented through vaccination are related to a higher willingness to receive the COVID-19 vaccine, because they seem to trigger the need of the vaccination to protect one’s health. This is in line with the results of two previous studies implying that the effects of different aspects of perceived control on medication adherence are mediated by necessity perceptions [57,58].

### 4.1. Practical Implications

The present findings provide evidence that the extended CSM is a useful framework in the context of illness-preventive behaviors. Illness representations about COVID-19 and perceptions about COVID-19 vaccination explained 69% of the variance in the willingness to receive the COVID-19 vaccine. Previous research showed that illness representations and perceptions about pharmaceuticals can be successfully changed by interventions and that these changes result in favorable behavior- and health-related outcomes [40,42,68,69,70]. Accordingly, changing relevant dimensions of illness representations and vaccination perceptions through public campaigns might contribute to increasing COVID-19 vaccination willingness among young adults. The development of such campaigns should be theory-guided, for example, by applying the Intervention Mapping (IM) approach [24], and include change techniques that are evidence-based [24,25].

Based on the findings of the present study and former research [19,64,65,66,67], the focus of these public campaigns should lie on increasing the perception that a COVID-19 infection can be prevented by vaccination and on fostering the perceived personal need of the vaccination for staying healthy, while concerns about side effects and long-term consequences of the vaccination should be decreased. According to previous literature [24,25], evidence-based change techniques that could be used to target these cognitions may be utilizing persuasive communication, modelling of the targeted behavior, introducing new arguments in favor of the behavior change, or stimulating anticipated regret. Additionally, the present results along with earlier findings [19,64,65,66,67] indicate that strengthening concerns about COVID-19 might also promote the willingness to receive the vaccine. However, Vollmann and colleagues [42] pointed out that as the illness representation dimensions perceived control and concerns are naturally negatively associated, it is fairly impossible to simultaneously threaten people and make them believe in an effective solution for a health problem in one intervention. On the other hand, fear as a result of perceived susceptibility and severity is an important motivator for health-promoting behavior. Therefore, risk perceptions may be targeted at the beginning of the intervention to make young adults aware of the health threat and thus raise concerns. Evidence-based change techniques that could be used here are raising consciousness of the consequences of the risk behavior or arousing fear by providing risk information. However, all these latter mentioned techniques should be combined with messages focusing on communicating effective control strategies and improving self-efficacy to reach optimal intervention effectiveness [24,71].

### 4.2. Limitations

The present study is subject to a number of weaknesses. First, because COVID-19 vaccines were not available for young adults in spring 2021, vaccination willingness has been investigated and not actual vaccination uptake. However, having a behavioral intention does not guarantee the actual realization of the behavior (intention–behavior gap) [72]. On the other hand, previous research showed that vaccination intention is a crucial predictor of actual vaccination uptake against influenza, HPV, and swine flu, explaining up to 58% of the variance in vaccination uptake [39,73,74]. These findings indicate that the willingness to receive the COVID-19 vaccine might translate to a reasonable extent into actual vaccination uptake. Second, due to the cross-sectional design, inferences about causality cannot be drawn [75]. Although the tested model is based on theoretical assumptions and empirical findings, it is also reasonable to assume that the interrelation of the variables is much more complex. For example, concerns about the safety of COVID-19 vaccination might lead to minimizing the seriousness of COVID-19 as a strategy of dissonance reduction [76]. Third, the present convenience sample limits the generalizability of the findings in different ways. In particular, the sample may be subject to self-selection bias since individuals with a more positive attitude towards vaccinations are more willing to participate in a study about vaccination. Furthermore, the participants were highly educated, which makes it likely that their health literacy was comparatively high [77], which is known to facilitate the understanding of medical information and the translation of this information into behavior that promotes and maintains health, such as vaccination willingness and uptake [78]. Finally, according to other established health behavior theories [79,80], illness representations and treatment perceptions are only two of many determinants of preventive behaviors. For example, reviews on vaccination hesitancy also identified self-efficacy, behavioral control, and social influences such as normative pressure as important psychological factors affecting the decision for or against vaccination [66,67,81]. Furthermore, contextual influences (e.g., access to health services, policies, communication and media environment) and vaccine-specific issues (e.g., costs, mode of administration, vaccination schedule) that have previously been identified as potential determinants in the context of vaccination [19,64,66,67,81] were not considered in the present study. Future studies would profit from investigating the actual COVID-19 vaccination uptake in a sample with a more heterogeneous educational background while considering multiple determinants of preventive behaviors and specifically vaccination uptake.

## 5. Conclusions

The extended Common Sense Model proved to be a useful framework in studying factors and processes underlying vaccination willingness among young adults. Moreover, it seems suitable for systematizing the numerous recent findings regarding psychological factors influencing COVID-19 vaccination willingness. Many of the current findings gained from rather explorative studies could be classified in the terms of the extended Common Sense Model in order to gain an integrative understanding of psychological determinants of COVID-19 vaccination willingness (e.g., [82]).

Furthermore, our findings underpin that prevention control is a relevant illness representation dimension in the context of illness-preventive behaviors. Public campaigns intending to increase the vaccination rate among young adults should aim at fostering perceptions about the effectiveness of vaccination to prevent COVID-19 and about the necessity of COVID-19 vaccination to stay healthy as well as at reducing concerns about negative side effects of COVID-19 vaccination.

## Figures and Tables

**Table 1:** Sample characteristics (*N* = 584).

Gender		Occupation	
female	408 (69.9%)	working	285 (48.9%)
male	167 (28.6%)	student/in training	285 (48.9%)
other	8 (1.4%)	homemaker	1 (0.2%)
no information	1 (0.2%)	unemployed	12 (2.1%)
**Highest education** **(ongoing or completed)**		**Higher risk of severe COVID-19 due to an underlying heath condition**	
secondary or vocational education	32 (5.5%)	yes	553 (94.7%)
tertiary education	552 (94.5%)	no	31 (5.5%)
**Previous COVID-19 infection (self)**		**Previous COVID-19 infection (others)**	
no	450 (77.1%)	no	89 (15.2%)
yes, but not tested	58 (9.9%)	yes	478 (81.8%)
yes, confirmed by a test	76 (13.0%)	not sure	17 (2.9%)

**Table 2:** Descriptives and bivariate correlations of the study variables.

	1	2	3	4	5	6	7	8	9	10	11	12	M (SD)
1 Vaccination willingness ^a^													8.10 (2.96)
**Illness representations ^a^**													
2 Identity	0.19 ***												4.49 (1.56)
3 Timeline	0.01	0.43 ***											3.47 (1.67)
4 Consequences	0.19 ***	0.30 ***	0.20 ***										6.30 (2.28)
5 Personal control	−0.15 ***	0.04	0.06	−0.01									4.10 (2.59)
6 Treatment control	0.27 ***	0.17 ***	0.07	0.16 ***	0.11 **								6.41 (2.18)
7 Prevention control through own behavior	0.30 ***	0.15 ***	0.05	0.14 **	0.06	0.23 ***							6.89 (1.86)
8 Prevention control through vaccination	0.76 ***	0.17 ***	0.05	0.15 ***	−0.10 *	0.32 ***	0.31 ***						7.65 (2.22)
9 Concern	0.42 ***	0.43 ***	0.30 ***	0.35 ***	−0.05	0.13 **	0.24 ***	0.38 ***					5.21 (2.39)
10 Emotional response	0.19 ***	0.18 ***	0.16 ***	0.17 ***	0.01	0.04	0.05	0.20 ***	0.40 ***				5.79 (2.31)
11 Understanding	0.08 *	0.03	−0.06	0.09 *	0.04	0.09 *	0.20 ***	0.12 **	0.03	−0.01			7.14 (1.70)
**Treatment perceptions ^b^**													
12 Necessity	0.76 ***	0.14 **	0.02	0.12 **	−0.11 **	0.27 ***	0.28 ***	0.82 ***	0.33 ***	0.18 ***	0.12 **		3.50 (1.19)
13 Concerns	−0.61 ***	−0.04	0.03	−0.03	0.10 *	−0.12 **	−0.19 ***	−0.53 ***	−0.16 ***	−0.07	−0.10 *	−0.57 ***	2.87 (1.29)

Note. ^a^ scale range 0–10; ^b^ scale range 1–5. * *p* < 0.05, ** *p* < 0.01, *** *p* < 0.001.

**Table 3:** Results of the multiple regression analyses.

Predictors	Outcome	
	Willingness to Receive the Vaccine	Perceptions of Necessity of the Vaccination
	ß	ß
**Illness representations**		
Identity	0.02	−0.01
Consequences	0.05	−0.02
Personal control	−0.06 *	−0.03
Treatment control	0.04	0.01
Prevention control through own behavior	0.04	0.03
Prevention control through vaccination	0.31 ***	0.79 ***
Concern	0.12 ***	0.02
Emotional response	−0.00	0.02
Understanding	−0.03	0.03
**Treatment perceptions**		
Necessity	0.29 ***	-
Concerns	−0.24 ***	-
	adj. *R*^2^ = 0.69 *F*(11,572) = 119.89 ***	adj. *R*^2^ = 0.66 *F*(9574) = 128.31 ***

Note. * *p* < 0.05, *** *p* < 0.001.

## Data Availability

The data presented in this study are available on request from the corresponding author, upon reasonable request.
